# Networking to Optimize *Dmd* exon 53 Skipping in the Brain of *mdx52* Mouse Model

**DOI:** 10.3390/biomedicines11123243

**Published:** 2023-12-07

**Authors:** Mathilde Doisy, Ophélie Vacca, Claire Fergus, Talia Gileadi, Minou Verhaeg, Amel Saoudi, Thomas Tensorer, Luis Garcia, Vincent P. Kelly, Federica Montanaro, Jennifer E. Morgan, Maaike van Putten, Annemieke Aartsma-Rus, Cyrille Vaillend, Francesco Muntoni, Aurélie Goyenvalle

**Affiliations:** 1 Université Paris-Saclay, UVSQ, Inserm, END-ICAP, 78000 Versailles, France; mathilde.doisy-caquant@uvsq.fr (M.D.); ophelie.vacca@uvsq.fr (O.V.); amel.saoudi@universite-paris-saclay.fr (A.S.);; 2 School of Biochemistry & Immunology, Trinity Biomedical Sciences Institute, Trinity College Dublin, D02 R590 Dublin, Ireland; cfergus@tcd.ie (C.F.);; 3 Dubowitz Neuromuscular Centre, UCL Great Ormond Street Institute of Child Health, 30 Guildford Street, London WC1N 1EH, UK; t.gileadi@ucl.ac.uk (T.G.); f.montanaro@ucl.ac.uk (F.M.); jennifer.morgan@ucl.ac.uk (J.E.M.); f.muntoni@ucl.ac.uk (F.M.); 4 National Institute for Health Research, Great Ormond Street Institute of Child Health Biomedical Research Centre, University College London, London WC1N 1EH, UK; 5 Department of Human Genetics, Leiden University Medical Center, 2333ZA Leiden, The Netherlands; m.a.t.verhaeg@lumc.nl (M.V.); m.van_putten@lumc.nl (M.v.P.); a.m.aartsma-rus@lumc.nl (A.A.-R.); 6 Université Paris-Saclay, CNRS, Institut des Neurosciences Paris-Saclay, 91400 Saclay, France; cyrille.vaillend@universite-paris-saclay.fr; 7 SQY Therapeutics-Synthena, UVSQ, 78180 Montigny le Bretonneux, France

**Keywords:** antisense oligonucleotides, exon skipping, Duchenne muscular dystrophy, brain comorbidities, exon 53, CNS delivery

## Abstract

Duchenne muscular dystrophy (DMD) is caused by mutations in the *DMD* gene that disrupt the open reading frame and thus prevent production of functional dystrophin proteins. Recent advances in DMD treatment, notably exon skipping and AAV gene therapy, have achieved some success aimed at alleviating the symptoms related to progressive muscle damage. However, they do not address the brain comorbidities associated with DMD, which remains a critical aspect of the disease. The *mdx52* mouse model recapitulates one of the most frequent genetic pathogenic variants associated with brain involvement in DMD. Deletion of exon 52 impedes expression of two brain dystrophins, Dp427 and Dp140, expressed from distinct promoters. Interestingly, this mutation is eligible for exon skipping strategies aimed at excluding exon 51 or 53 from dystrophin mRNA. We previously showed that exon 51 skipping can restore partial expression of internally deleted yet functional Dp427 in the brain following intracerebroventricular (ICV) injection of antisense oligonucleotides (ASO). This was associated with a partial improvement of anxiety traits, unconditioned fear response, and Pavlovian fear learning and memory in the *mdx52* mouse model. In the present study, we investigated in the same mouse model the skipping of exon 53 in order to restore expression of both Dp427 and Dp140. However, in contrast to exon 51, we found that exon 53 skipping was particularly difficult in *mdx52* mice and a combination of multiple ASOs had to be used simultaneously to reach substantial levels of exon 53 skipping, regardless of their chemistry (tcDNA, PMO, or 2′MOE). Following ICV injection of a combination of ASO sequences, we measured up to 25% of exon 53 skipping in the hippocampus of treated *mdx52* mice, but this did not elicit significant protein restoration. These findings indicate that skipping mouse dystrophin exon 53 is challenging. As such, it has not yet been possible to answer the pertinent question whether rescuing both Dp427 and Dp140 in the brain is imperative to more optimal treatment of neurological aspects of dystrophinopathy.

## 1. Introduction

Duchenne muscular dystrophy (DMD) is an X-linked genetic disorder characterized by progressive muscle degeneration and weakness, affecting approximately 1 in every 5000 live male births. While the peripheral manifestations of DMD have been extensively studied, the associated brain comorbidities represent a critical yet poorly understood facet of the disease. Brain comorbidities in DMD encompass a spectrum of neurological comorbidities, including cognitive impairment, learning disabilities, attention deficits, and emotional dysregulation [1,2,3,4]. These complex manifestations can significantly impact the overall quality and participation in life for affected individuals. Recent advancements in therapeutic innovation for DMD have resulted in the conditional approval of several treatment modalities, including antisense oligonucleotide (ASO)-mediated exon skipping and gene therapy approaches [5]. These interventions mark pivotal strides in mitigating the striated muscle manifestations of the disease, offering hope for enhanced muscle and cardiac function and improved life expectancy. However, none of these therapies yet have addressed brain comorbidities. With the potential for improved life expectancy now on the horizon, the imperative to tackle DMD neurological challenges gains even greater significance.

The European BIND consortium stands as a collaborative effort that aims to address the multifaceted challenges posed by brain comorbidities in DMD (https://bindproject.eu/ accessed on 1 September 2023). Bringing together experts from diverse fields including genetics, neurology, and therapeutic development, the consortium strives to unravel the role of dystrophins in the brain and hopefully pave the way for innovative treatments that can improve the quality of life of DMD patients.

The *mdx52* mouse model, representative of a substantial portion of DMD patients with regards to mutation type and location [6,7], offers a valuable platform for investigating therapeutic interventions aimed at addressing both peripheral and central aspects of the disorder [8]. Interestingly, the *mdx52* mouse model presents a unique opportunity for exon skipping therapies as it is amenable to exon 51 skipping as well as exon 53 skipping, two strategies that hold the potential to restore the expression of functional dystrophins and for which ASOs have received accelerated approval by the FDA. Furthermore, this mutation affects both the full-length dystrophin (Dp427) that is lacking in skeletal muscle and brain from all DMD patients, but also brain Dp140, which is lacking in about 50% of DMD patients [6]. Dp140 has an isoform-specific promoter in intron 44, followed by a long 5′ UTR and a start codon in exon 51. This isoform is exclusively expressed in the brain. Several studies have found that patients lacking Dp140 and full-length dystrophin have a higher risk of brain comorbidities [4,9,10,11,12].

Previous work from the BIND consortium highlighted the effectiveness of exon 51 skipping in the *mdx52* adult mouse brain [13,14]. We demonstrated that this treatment led to partial restoration of the Dp427 isoform and yielded improvements in behavioral deficits. However, the specificity of exon 51 skipping restricts its impact solely to Dp427 restoration, leaving the Dp140 isoform absent due to its reliance on exon 51 for the start codon. In contrast, exon 53 skipping offers the opportunity to restore both Dp427 and Dp140 isoforms. In this study, we embarked on the exploration and optimization of exon 53 skipping in the brain of *mdx52* mice. We first screened ASOs targeting the mouse *Dmd* transcript in vitro in control and *mdx52* myotubes that express higher levels of *Dmd* transcripts than neuronal cell lines, using various chemistries of ASOs including tricyclo-DNA (tcDNA), phosphorodiamidate morpholino oligomer (PMO), and 2′-methoxyethyl phosphorothioate (2′MOE). We show here that regardless of the ASO chemistry, substantial skipping of exon 53 necessitates the design and combination of multiple ASOs. The combination of two or three ASOs was able to skip up to 30% of exon 53 in vitro. This was confirmed in vivo after intramuscular injection in *mdx52* mice. Encouraged by these findings, we shifted to the central nervous system and performed ICV injections as previously optimized [14]. Notably, the combination of three ASOs demonstrated substantial levels of exon 53 skipping in the cerebellum, hippocampus, and cortex.

Yet, despite achieving efficient exon 53 skipping in the *mdx52* mouse model, quantification of protein expression revealed only minimal levels of restoration, rendering any investigation into behavioral enhancement futile.

## 2. Materials and Methods

*Cell Culture and Transfection.*

*H2K-mdx52* myoblasts [15] were grown at 33 °C in DMEM medium containing γ-IFN at a concentration of 20 units/mL and 20% (*vol*/*vol*) FBS in 10% CO_2_. To induce differentiation of proliferating myoblasts to myotubes, the cells were grown in differentiation medium containing 5% (*vol*/*vol*) horse serum at 37 °C in 5% CO_2_.

Primary-*mdx52* myoblasts were obtained from dissection of skeletal muscles of 2-week-old *mdx52* mice. For isolation of myoblasts, the Skeletal Muscle Dissociation Kit (MACS, Miltenyi Biotec, Bergisch Gladbach, Germany) was used. Cells were grown at 37 °C in DMEM medium containing Ultroser G (Sartorius, Göttingen, Germany) at 2% (*vol*/*vol*) and 20% (*vol*/*vol*) FBS in 5% CO_2_. After the treatment, the cells were grown in differentiation medium containing 5% (*vol*/*vol*) horse serum at 37 °C in 5% CO_2_.

C2C12 myoblasts [16] were grown at 37 °C in DMEM medium containing 10% (*vol*/*vol*) FBS at 37 °C in 10% CO_2_. After the treatment, the cells were grown in differentiation medium containing 2% (*vol*/*vol*) FBS at 37 °C in 10% CO_2_.

**Figure 1:**
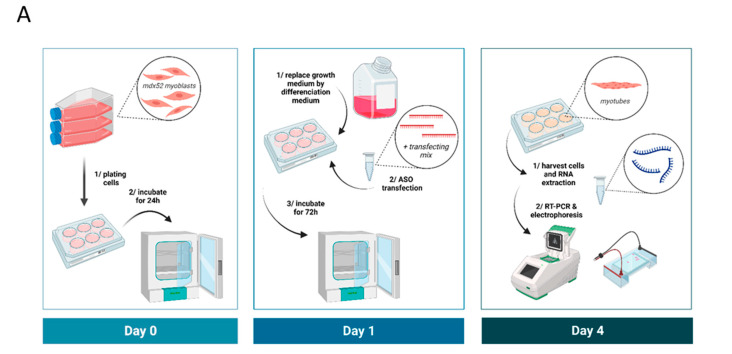

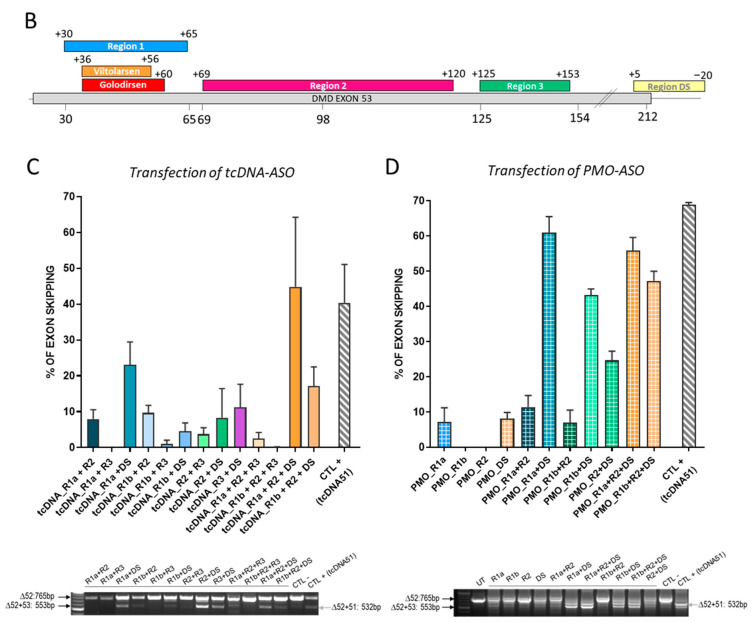
In vitro evaluation of combination of selected tcDNA and PMO ASOs. (**A**) Schematic representation of the in vitro analysis workflow. (**B**) Localization of the selected ASO in comparison with golodirsen and viltolarsen sequences. (**C**) Quantification of exon 53 skipping levels by RT-PCR after transfection of *H2K-mdx52* myotubes with different combinations of 20-mer tcDNA targeting mouse exon 53. Results are expressed as means ± SEM; *n* = 4 transfections. CTL-: no ASO transfected. The level of exon 51 obtained after the transfection of the previously described tcDNA51 is shown as a positive control of transfection (CTL+). Exon 51 skipping level of 40%. The combinations that induce the best exon 53 skipping are R1a + DS (23%), R1a + R2 + DS (45%), and R1a + R2 + R3 + DS (33%). A representative gel of electrophoresis is shown below the graph. (**D**) Quantification of exon 53 skipping levels by RT-PCR after transfection of primary *mdx52* myotubes with different combinations of PMO targeting mouse exon 53. Results are expressed as means ± SEM; *n* = 3 transfections. The level of exon 51 obtained after the transfection of tcDNA51 is shown as a positive control of transfection (CTL+). Exon 51 skipping level of 69%. The combinations that induce the best results are R1a + DS (61%), R1b + DS (43%), R1a + R2 + DS (56%), and R1b + R2 + DS (47%).

TcDNA-ASOs were transfected into cells using the Lipofectamine LTX (Thermo Fisher Scientific, Rockford, IL, USA) according to the manufacturer’s instructions. Briefly, 24 h before transfection, cells were plated in 6-well plates (1.10^5^ cells per well). The next day, cells were put in differentiation medium and transfected with 100 nM of tcDNA and Lipofectamin LTX reagent. After transfection, cells were incubated for 72 h in differentiation medium before being collected in TRIzol reagent to isolate total RNA, according to the manufacturer’s instructions (Thermo Fisher Scientific, Rockford, IL, USA). A workflow of the transfection analysis is represented in Figure 1A.

PMOs were transfected into cells using Endoporter (Genetools LC, Philomath, OR, USA). Primary *mdx52* myoblasts were plated into 12-well plates (75,000 cells per well) 24 h before transfection. The growth medium was replaced with differentiation medium along with 10 µM PMO and 3 µL Endoporter per well. Cells were incubated for 72 h before collection in TRIzol reagent to isolate RNA.

For 2′MOE transfections, C2C12 myoblasts were cultured at 37 °C in DMD medium containing 10% FBS, 1% P/S, 2% Glutamax, and 1% glucose and kept at 10% CO_2_. Cells were differentiated for 7 days using differentiation medium (DMEM with 2% FBS, 1% P/S, 2% Glutamx, and 1% glucose) before transfection. Up to 200 nM of 2′MOE was added to the cells with Lipofectamine 2000 (Thermo Fisher Scientific, Rockford, IL, USA). After 48h, the cells were harvested in TRIsure for RNA isolation according to the manufacturer’s instruction (Meridian Bioscience, London, UK).

*Animals and antisense oligonucleotides.*

Exon 52-deleted X chromosome-linked muscular dystrophy mice (*mdx52* mice) were generated by the group of Dr. Katsuki Motoya [8]. Exon 52 of the *DMD* gene was replaced with a neomycin resistance gene, thereby eliminating expression of Dp427, Dp260, and Dp140 dystrophins, but preserving expression of Dp116 (in peripheral nerves) and of Dp71 (in brain and retina) [8]. The mouse line was backcrossed to C57BL/6J for more than eight generations. The mouse line was provided by Prof. Sasaoka Toshikuni (Department of Comparative & Experimental Medicine/Brain Research Institute; Niigata University, Japan). Breeders were provided to this study by Dr. Jun Tanihata and Dr. Shin’ichi Takeda (National Center of Neurology and Psychiatry, Tokyo, Japan). Heterozygous females were crossed with C57BL/6JRj males to generate *mdx52* and littermate control (WT) males at the animal facility Plateforme 2Care, UFR des Sciences de la santé, Université de Versailles-Saint Quentin (France). At Trinity College Dublin Transgenic facility, heterozygous females were crossed to C57BL/6J males to generate *mdx52* and littermate control (WT) males. Genotypes were determined by PCR analysis of ear or tail DNA. Mice were housed in individually ventilated cages in a specific pathogen-free facility on a 12 h light/dark cycle with access to food and water ad libitum. Animal care and all experimental procedures complied with the national and European legislation, the ARRIVE guidelines, and were approved by the French government (Ministère de l’Enseignement Supérieur et de la Recherche, Autorisation APAFiS #6518) and the Irish Health Products Regulatory Authority (Ref: AE19136/P131) and with the approval of the TCD Animal Research Ethics Committee.

The tcDNA-ASOs used in this study were synthesized by SQY Therapeutics (Montigny-le-Bretonneux, France) and were full phosphodiester and conjugated at their 5′end via a C6-amino linker to palmitic acid as previously described [17]. PMO-ASOs were purchased from Genetools LLC (Philomath, OR, USA) and 2′MOE were purchased from Eurogentec (Seraing, Belgium).

*Intramuscular and intracerebroventricular injections.*

Intramuscular injections (IM) were performed in the *Tibialis anterior* (TA) of adult *mdx52* mice anesthetized under isoflurane (2.5%, 150–250 mL/min). A volume of 30 µL was injected per TA containing tcDNA-ASO (100 µg) or PMO-ASO (50 µg).

ICV injections were performed in 6–8-week-old *mdx52* mice deeply anesthetized by a single intraperitoneal injection of ketamine (95 mg/kg)/medetomidine (1 mg/kg). ASOs in saline solution (phosphate-buffered saline, 0.1 mol/L) were injected bilaterally into the lateral brain ventricles (−0.5 mm from bregma; 1 mm lateral; −2 mm from dura) [18]. A volume of 5 μL was infused in each ventricle at a rate of 0.3 μL/min. A total amount of 400 µg of tcDNA or 900 µg of PMO was thus distributed bilaterally. For the combinations of several ASOs, a mix containing an equimolar amount of the various ASOs was prepared to reach a total of 400 µg for tcDNA and 900 µg for PMO.

The animals were euthanized at different time points as indicated in the results section and brains were dissected out to isolate hippocampus (HIP), cerebellum (CBL), and cortex (CX) that were snap-frozen in liquid nitrogen for RNA and protein analysis.

*RNA Analyses.*

Total RNA was isolated from dissected brain structures, muscles, or cells using TRIzol reagent according to the manufacturer’s instructions (ThermoFisher Scientific, Rockford, IL, USA). For visualization of exon skipping efficacy on gels, aliquots of 1 µg of total RNA were used for RT-PCR analysis using the LunaScript RT SuperMix Kit (New England Biolabs, Evry, France), in a 20 μL reaction. The cDNA synthesis was carried out at 55 °C for 10 min. Next, the PCR was performed from 1.5 µL of cDNA with GoTaq G2 Colorless Master Mix (Promega, Madison, WI, USA) in a 25-µL reaction using the primers Ex-m50F (5′-AGGAAGTTAGAAGATCTGAGG -3′) and Ex-m55R (5′- GGAACTGCTGCAGTAATCTATGA-3′) and the following cycling conditions: 32 cycles of 95 °C (30 s), 58 °C (1 min) and 72 °C (1 min). PCR products were electrophoresed on 1.5% agarose gels and quantified with ImageLab Software version 6.1 (Bio-Rad, Marnes-la-coquette, France).

Exon 53 skipping was also measured by Taqman quantitative PCR as described [19], using Taqman assays designed against the exon 53–54 junction (assay Mm.PT.58.41709800: Forward: 5′-AGAAGGTCCTCACACAGTAGA-3′; reverse: 5′-CAGCAGAATAGTCCCGAAGAAG-3′; Probe: 5′-CGGCAGATAAGTGTAGACGTGGCA-3′) and exon 51–54 junction (Forward: 5′-TCTTTGGCCAACTGCTTCT-3′; reverse: 5′-CGAGCTTGGACAGAACTTACA-3′; Probe: 5′-AGAGAGTGATGGTGGGTGATCTGGA-3′) (Integrated DNA technology, Leuven, Belgium). As input, 150 ng of cDNA was used per reaction and all assays were carried out in triplicate. Assays were performed under fast cycling conditions on a Biorad CFX384 Touch Real-Time PCR Detection System, and all data were analyzed using the absolute copy number method. For a given sample, the copy number of skipped products (exon 53–54 assay) and unskipped products (exon 51–54 assay) were determined using the standards Ex49–54Delta52 and Ex48–55Delta52+51, respectively (gBlocks gene fragments from Integrated DNA technology, Leuven, Belgium). Exon 53 skipping was then expressed as a percentage of total dystrophin transcripts (calculated by the addition of exon 53–54 and exon 51–54 copy numbers).

*Western blot analyses.*

Protein extracts were obtained from brain structures treated with RIPA lysis and extraction buffers (Thermo Fisher Scientific, Rockford, IL, USA) complemented with SDS powder (5% final) (Bio-Rad, Marnes-la-coquette, France ). Total protein concentration was determined with the BCA Protein Assay Kit (Thermo Fisher Scientific, Rockford, IL, USA). Samples were denatured at 100 °C for 3 min and 25 μg of protein was loaded onto NuPAGE 3–8% Tris-Acetate Protein gels (Thermo Fisher Scientific, Rockford, IL, USA), following manufacturer instructions. Dystrophin protein was detected by probing the membrane with the AB154168 rabbit primary monoclonal antibody (AB154168, Abcam, France) and vinculin was detected as an internal control with the hVin-1 primary antibody (Sigma, Saint-Louis, MI, USA), followed by incubation with a goat anti-mouse secondary antibody for vinculin (IRDye 800CW Goat anti-mouse IgG, Li-Cor, Bad Homburg, Germany) and a goat anti-rabbit for dystrophin (IRDye 700CW Goat anti-rabbit IgG, Li-Cor, Bad Homburg, Germany). Bands were visualized using the Odyssey CLx system (Li-Cor, Bad Homburg, Germany). Quantification was performed using the Empiria Studio software version 3.0 (Li-Cor, Bad Homburg, Germany) after normalization to internal control (vinculin) and based on a standard curve specific for each brain structure and made of a mix of WT and *mdx52* control lysates to obtain defined percentages of dystrophin (0, 5, 10, and 20% of corresponding WT tissues).

*Statistical analysis.*

Data are presented as means ± SEM; statistics were performed using the GraphPad Prism8 software (San Diego, CA, USA). All data that passed the normality test (Shapiro–Wilk normality test) were analyzed using standard one-way or two-way (for group comparisons) analyses of variance (ANOVAs). A Kruskal–Wallis test followed by Dunn’s post hoc multiple comparison were used to analyze data that did not pass the normality test. Group comparisons were performed using two-way ANOVAs with repeated measures (RM) when needed (for example different brain structure from the same animal), followed by Tukey (for one-way ANOVA) or Holm–Sidak (for two-way ANOVA) post hoc multiple comparisons. The significance threshold was set at *p* < 0.05.

## 3. Results

### 3.1. In Vitro Screening of ASO Sequences Targeting Mouse Dmd Exon 53

Considering that two ASOs, golodirsen (25-mer +36+60) and viltolarsen (21-mer +36+56), have already been approved by the FDA to skip the human exon 53, we first started by targeting the same region of the mouse *Dmd* exon 53. We initially designed a series of 12 tcDNA-ASOs overlapping this region (from +27 to +87) (see list of all ASOs in Table 1) and tested their ability to skip exon 53 in vitro in *H2K-mdx52* myotubes. Seventy-two hours after tcDNA-ASO transfection, cells were collected and RNA analyzed by RT-PCR with primers targeting exons 50 and 55 (workflow represented in Figure 1A).

In contrast with a positive control, tcDNA targeting exon 51, which had previously demonstrated skipping efficacy in the brain of *mdx52* mice [13,14] and which induced efficient exon 51 skipping in vitro (~50%), none of the exon 53-targeting tcDNA was able to induce more than 15% of exon skipping (Supplementary Figure S1A). We, therefore, designed a new series of longer tcDNA-ASOs (20-mer) targeting different regions of the murine exon 53 that we named Region 1 (+30+65), Region 2 (+69+120), Region 3 (+125+153), and Region DS (+5−20) (Figure 1B). We selected these regions based on previously described work [20], but also for their enrichment in exonic splicing enhancers (ESE) (Supplementary Figure S1B). Transfection of each single ASO in *H2K-mdx52* cells revealed, again, no more than 15% of exon 53 skipping (Supplementary Figure S1C). We then selected the best ASO sequence in each region to investigate the efficacy of different combinations of sequences. For region 1, we selected two ASO sequences, tcDNA +36+55 and +45+64, that we named tcDNA-R1a and R1b, respectively; for region 2, we selected the tcDNA +72+91 that we named tcDNA-R2; for region 3 we selected the tcDNA +131+150 that we named tcDNA-R3; and finally for region DS, we selected the tcDNA +5−15 that we named tcDNA-DS (Supplementary Figure S1C and Table 1).

Transfection of combination of two or three tcDNA-ASOs targeting exon 53 induced significantly higher exon skipping than transfection of a single ASO in *H2K-mdx52* myotubes (Figure 1C). The combination of two tcDNA-ASOs that induced the highest exon 53 skipping was R1a + DS and even higher levels could be obtained after the transfection of three tcDNA-ASOs, R1a + R2 + DS (~44% of exon 53 skipping). In order to check that this phenomenon was not chemistry-dependent, we next investigated whether combinations of ASOs made of a different chemistry, namely the phosphorodiamidate morpholino (PMO), would also induce higher skipping levels than single transfection. PMO-ASOs were designed to target similar sequences on exon 53 (Table 1) and were transfected into primary *mdx52* myotubes. The PMO-ASOs target slightly longer sequences (25-mer) at these regions, with PMO-R1a targeting +36+60 (the same target region of Golodirsen in human), PMO-R1b targeting +44+68, and PMO-DS targeting +9−16. As previously observed for transfection of single tcDNA-ASOs, transfection of single PMO-ASOs in *mdx52* myotubes led to poor exon 53 skipping. We detected higher levels of exon 53 skipping following transfection of two or three PMO sequences (Figure 1D) with R1a + DS reaching ~60% and R1a + R2 + DS reaching 55%. Similar results were obtained when transfecting the C2C12 cell line with the same PMO-ASOs (Supplementary Figure S2), although overall skipping efficiency was lower in this cellular model.

Interestingly, the same combination of two ASOs (R1a + DS) or three ASOs (R1a + R2 + DS) were the best for both tcDNA and PMO ASOs. Similar findings were obtained with a third chemistry, the 2′MOE, confirming that exon 53 skipping could only be induced at significant levels when several sequences were simultaneously transfected, regardless of the chemistry (Supplementary Figure S3).

### 3.2. Intramuscular Injection of Single or Combined ASO Targeting Exon 53 in mdx52 Mice

**Figure 2:**
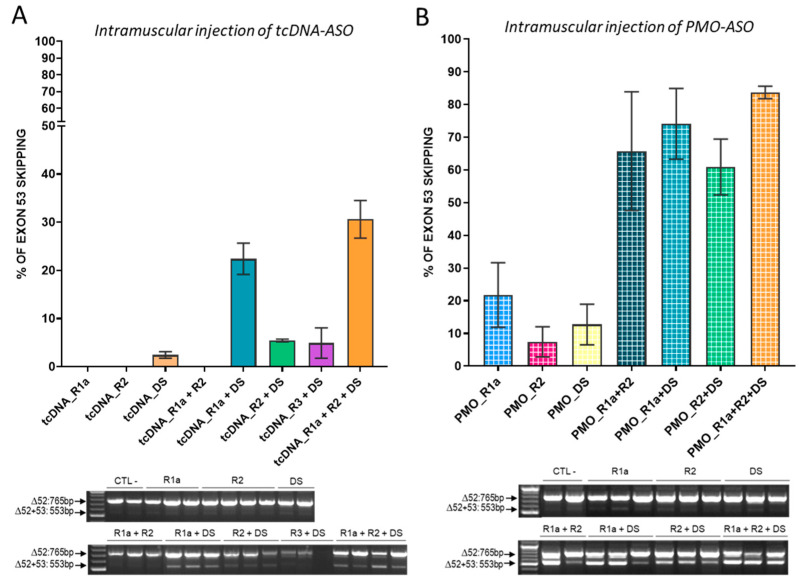
Intramuscular injections of single or combined ASOs targeting exon 53 in *mdx52* mice (**A**) Quantification of exon 53 skipping levels by RT-qPCR after intramuscular injections (IM) of tcDNA53-combinations in tibialis anterior muscle (TA) of *mdx52* mice. Results are expressed as means ± SEM; *n* = 3–6 TA analyzed 3 weeks after injection. (**B**) Quantification of exon 53 skipping levels by RT-qPCR after IM injection of PMO53-combination in TA of *mdx52* mice. Results are expressed as means ± SEM; *n* = 3 TA analyzed 2 weeks after injection. CTL-: no ASO transfected.

We next evaluated the extent of exon 53 skipping that we could obtain in vivo by injecting the ASO alone or in combination in the tibialis anterior muscle of *mdx52* mice. Adult *mdx52* mice were injected intramuscularly with a total of 100 µg of tcDNA (either alone or combined) and muscles were harvested 3 weeks later for RNA analysis. RT-qPCR revealed that treatment with single tcDNA ASOs induced only very low levels of exon 53 skipping (<2%) whereas treatment with two or three tcDNAs induced up to 30% exon skipping (Figure 2A). Similarly, intramuscular injection of PMO-ASO indicated that a combination of PMO induced much higher exon skipping (up to 85%) than single PMO (<20%) (Figure 2B). Altogether, these in vivo results confirmed that the combinations of ASOs inducing the highest level of exon 53 skipping were R1a + DS and R1a + R2 + DS.

### 3.3. Intracerebroventricular Injection of Combined ASOs Targeting Exon 53 in mdx52 Mice

**Figure 3:**
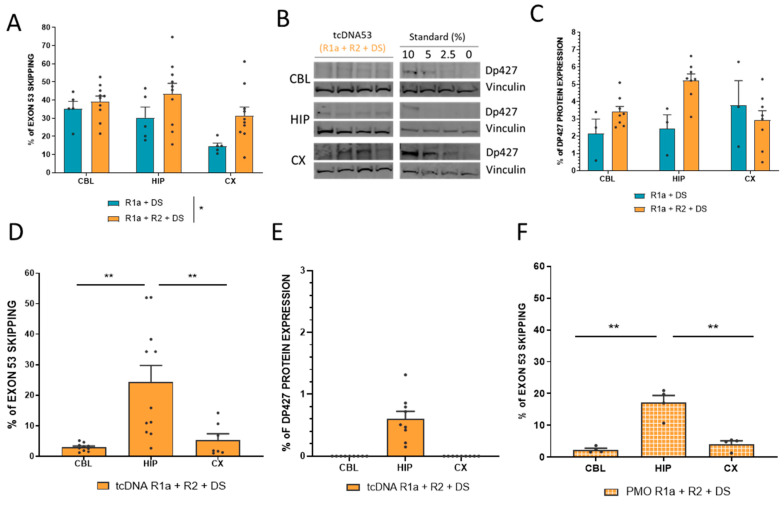
Intracerebroventricular injection of combined ASOs to skip mouse Dmd exon 53 in the brain of *mdx52* mice. (**A**) Quantification of exon 53 skipping levels by RT-qPCR in various CNS tissues (CBL: cerebellum, HIP: hippocampus, and CX: cortex) 4 weeks post-ICV administration of 2 (R1a + DS) or 3 (R1a + R2 + DS) tcDNA-ASOs. Results are expressed as means ± SEM; *n* = 5 mice for R1a + DS and *n* = 10 mice for R1a + R2 + DS. * *p* = 0.0383 between R1a + DS and R1a + R2 + DS, analyzed via RM two-way ANOVA. (**B**) Representative Western blots and (**C**) Quantification of Dp427 protein restoration 4 weeks post-ICV administration (means ± SEM; *n* = 3 R1a + DS and *n* = 8 R1a + R2 + DS). Immunoblots shown in (**B**) are representative examples of dystrophin restoration in the CBL, HIP, and CX. A 4-point standard curve made of 0, 2.5, 5, and 10% of WT lysate (mixed with *mdx52* lysate) was loaded for quantification. Vinculin was used as a control for normalization. (**D**) Quantification of exon 53 skipping levels by RT-qPCR in CBL, HIP, and CX 10 weeks post-ICV injection of tcDNA R1a + R2 + DS. Results are expressed as means ± SEM; *n* = 11 mice. ** *p* < 0.01 between CBL and HIP and between HIP and CX, analyzed via one-way ANOVA (**E**) Quantification of Dp427 protein restoration 10 weeks post-ICV administration (means ± SEM; *n* = 9 mice per group. (**F**) Quantification of exon 53 skipping levels by RT-qPCR 8 weeks post-ICV administration of PMO R1a + R2 + DS. Results are expressed as means ± SEM; *n* = 4 mice per group. ** *p* < 0.01 between CBL and HIP and between HIP and CX, analyzed via one-way ANOVA.

Based on the in vitro and intramuscular results, we selected the two best combinations of tcDNA-ASOs (R1a + DS and R1a + R2 + DS) to evaluate their ability to skip exon 53 in the brain of mdx52 mice. For this experiment, 8-week-old *mdx52* mice received ICV injections of a total of 400 µg of tcDNA, as previously described for exon 51, using a tcDNA-Ex51 [13]. In each case, an equimolar mixture of ASOs was injected, i.e., 200 µg of each for R1a + DS combination and 133 µg of each for R1a + R2 + DS. Exon skipping levels were examined 4 weeks after the injection in different regions of the CNS (CBL, HIP, CX). We quantified the efficacy of exon 53 skipping in the different brain regions by real-time TaqMan PCR quantification. The ICV injection of the tcDNA combination R1a + DS induced a mean of 27% of exon 53 skipping across the various structures while the combination R1a + R2 + DS induced significantly higher levels of exon 53 skipping with a mean of 38% (treatment effect *p* = 0.0383, analyzed via RM two-way ANOVA) (Figure 3A). We then quantified the restoration of the dystrophin protein (Dp427) by Western blot in CBL, HIP, and CX, and detected only low levels of restoration 4 weeks after the injection, with a mean of 2.8% and 3.8% of restoration for combination R1a + DS and R1a + R2 + DS, respectively (treatment effect *p* = 0.1294 analyzed via RM two-way ANOVA) (Figure 3B).

We previously showed for exon 51 skipping that protein restoration was higher between 7 and 11 weeks post-injection in the CNS [13], suggesting that the 4-week time point post-injection may not be optimal to assess protein recovery. We, therefore, injected a new group of mice with the combination of tcDNA R1a + R2 + DS, inducing the highest skipping and protein restoration levels for analysis at a later time point. The different brain regions were analyzed 10 weeks after the ICV injection and quantification of exon 53 skipping revealed significantly lower levels of exon 53 skipping than those detected 4 weeks after the injection (treatment effect *p* < 0.0001 between 4 weeks and 10 weeks, analyzed via RM two-way ANOVA) (Figure 3D). The levels were particularly lower in CBL and CX compared to HIP (*p* = 0.0011 and *p* = 0.0084, respectively, analyzed via one-way ANOVA), which was not observed at the 4-week time point. We next assessed the levels of protein restoration in these brain regions by Western blot and found extremely low levels of Dp427 restoration in HIP (<1%) and could not detect any protein restoration in CBL and CX (Figure 3E). This was also confirmed using a capillary Western immunoassay with Jess (Supplementary Figure S4A). Similarly, we could not detect any increase in Dp140 protein (Supplementary Figure S4B).

To investigate whether another chemistry of ASO would lead to a different outcome, we also injected the best combination R1a + R2 + DS as PMO. The 6-week-old *mdx52* mice received ICV injections of a total 900 µg of PMO as previously described for exon 51 [14]. The PMO mixture was composed of equivalent quantity of each PMO of the combination, i.e., 300 µg of each. Exon skipping levels were quantified in CBL, HIP, and CX by real-time TaqMan PCR 8 weeks after the injection. The levels of exon 53 skipping were slightly lower than those obtained with tcDNA, but otherwise followed a very similar pattern of distribution across the three different brain regions, with higher skipping levels in HIP (~17%) compared to CBL and CX (2.3% and 4.2%, respectively) (Figure 3F). Similar to the tcDNA-injected brains, Dp427 was barely detectable in PMO-injected brains (Supplementary Figure S5).

### 3.4. Combination of four ASOs Targeting Exon 53 Skipping Achieves Higher Level of Exon Skipping but Still Only Very Low Levels of Protein Restoration

**Figure 4:**
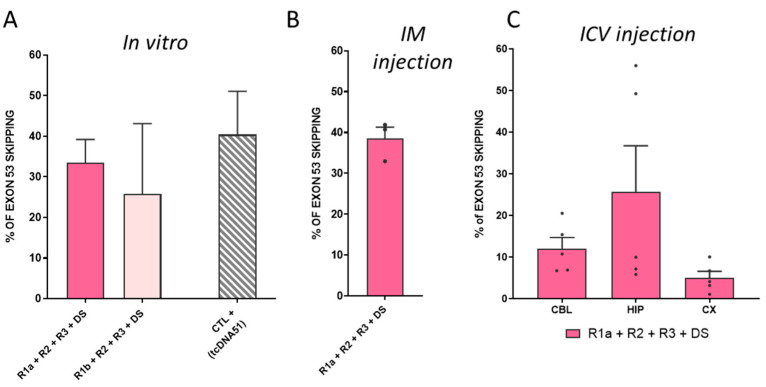
Combining 4 tcDNA-ASOs induces high level of exon 53 skipping after intracerebroventricular injections in *mdx52* mice (**A**) Quantification of exon 53 skipping levels by RT-PCR after transfection of *H2K mdx52* cells with different combinations of 20-mer tcDNA53. Results are expressed as means ± SEM; *n* = 3–4 transfections compared to tcDNA51 exon 51 skipping level (CTL+ 40%). The combination that induces the highest exon 53 skipping is R1a + R2 + R3 + DS (33%). (**B**) Quantification of exon 53 skipping levels by RT-qPCR after intramuscular injections (IM) of tcDNA53-combination in tibialis anterior muscle (TA) of *mdx52* mice. Results are expressed as means ± SEM; *n* = 3 TA analyzed 3 weeks after injection. R1a + R2 + R3 + DS (38%). (**C**) Quantification of exon 53 skipping levels by RT-qPCR after ICV in CBL, HIP, and CX 10 weeks after tcDNA53 ICV administration. Results are expressed as means ± SEM; *n* = 5 mice.

In an attempt to increase levels of exon 53 skipping and protein restoration, we investigated the combination of up to four ASO sequences, corresponding to the four regions initially identified, i.e., R1, R2, R3, and DS (Supplementary Figure S1). We transfected H2K-*mdx52* myotubes with two different tcDNA-ASO combinations: R1a + R2 + R3 + DS and R1b + R2 + R3 + DS. In line with what was observed with transfection of three ASOs, R1a was better than R1b and the transfection of R1a + R2 + R3 + DS yielded higher levels of exon 53 skipping, reaching up to 34%, which was in the same range as the levels of exon 51 skipping that we obtained with the positive control tcDNA-Ex51 (Figure 4A). We next injected this combination of four tcDNA-ASOs intramuscularly in the tibialis anterior of *mdx52* mice and detected up to 39% of exon 53 skipping 3 weeks after the injection (Figure 4B). Finally, we injected this combination by ICV administration in the brain of *mdx52* mice and measured an average of 14% of exon 53 skipping across the three structures (12% in CBL, 26% in HIP and 5% in CX), which was slightly higher than the levels obtained with the combination of R1a + R2 + DS, although not statistically different (treatment effect *p* = 0.4339, between the two combinations analyzed via two-way RM ANOVA) (Figure 4C). Despite the slightly higher levels of exon 53 skipping, we did not detect higher protein restoration and failed to detect any Dp140 restoration as previously with the combination of three ASOs (Supplementary Figure S6).

## 4. Discussion

In this study, we embarked on the evaluation and optimization of exon 53 skipping with the aim to restore both Dp427 and Dp140 expression within the brains of *mdx52* mice. We previously demonstrated that *mdx52* mice display more severe phenotypes than *mdx* mice that only lack Dp427, in particular, enhanced anxiety and a severe impairment in an amygdala-dependent Pavlovian associative fear learning and memory task [21], suggesting that the combined deficiency of Dp427 and Dp140 leads to a more severe brain phenotype. We also showed that skipping exon 51 in the brains of *mdx52* mice resulted in restoration of Dp427 (5–15% of wild-type levels), which was associated with a partial improvement of the central deficits [13]. One of the hypotheses to explain the incomplete rescue of behavioral outcomes in this model was the possibility that the restoration of solely Dp427, leaving Dp140 absent, was not sufficient. Indeed, because exon 51 contains the start codon of Dp140, this isoform cannot be rescued following exon 51 skipping. In contrast, skipping exon 53 should allow the rescue of a slightly shorter (Δ52–53) but functional Dp140 in addition to Dp427. Consequently, we targeted exon 53 of the mouse *Dmd* transcript, initially with tcDNA-ASO that had demonstrated superiority in our previous optimization studies focused on exon 51 skipping [14]. However, we quickly realized that skipping the mouse exon 53 was much more challenging than skipping the human exon 53. We thus performed further screening, also using different chemistries of ASO to demonstrate that this was not a chemistry-dependent issue. This low skipping efficacy in mouse cells was surprising as two PMOs have already been conditionally approved for exon 53 skipping in DMD patients (golodirsen and viltolarsen) demonstrating in humans a similar level of exon skipping to eteplirsen, targeting exon 51. However, targeting similar sequences on the mouse *Dmd* exon 53 was inefficient and we had to design and combine multiple ASOs in order to achieve substantial exon 53 skipping. This disparity between human and mouse exon 53 skipping potential raises intriguing questions. We could hypothesize that the splicing regulation of this particular exon is different in mice and humans. Based on the analysis performed with the Human Splicing Finder system (https://hsf.genomnis.com), the two exons appear to share a lot of similarities in regulatory signals (Supplementary Figure S7). The only main difference detected is the presence of a cryptic donor splice site in mouse exon 53 (at position 134). This may actually explain the intermediate band that was occasionally detected by RT-PCR and which corresponds to a partial skipping of exon 53. This elimination of only 78 nt from exon 53 was also observed by Mitrpant and colleagues in a previous study in wild-type mice [20]. The same authors reported the skipping of both exon 53 and 54 following treatment with some ASOs, in particular when targeting region +39+89, but we did not observe this phenomenon (even when amplifying a larger fragment, e.g., between exon 50 and 58). The apparent divergence in exon 53 skipping between the two species remains an enigma, warranting further investigations.

Despite being challenging, exon 53 skipping was achieved at substantial levels when several ASOs were used, highlighting the significance of targeting multiple regions within an exon to enhance skipping efficiency. This pattern was evident across various chemistries and supported by both in vitro and in vivo studies, although it is worth mentioning that not all combinations yielded higher skipping efficacy. Previous work has also shown that combining ASOs can induce more efficient skipping of a single exon [22,23] or can even be used to successfully achieve multi-exon skipping [24,25]. The cumulative evidence from our study, together with previous ones, confirms the notion that several ASOs end up in the same nucleus after uptake, thus facilitating cooperative exon skipping.

The comparison between tcDNA and PMO chemistries targeting exon 53 in this work confirms the trend observed in our previous study focusing on exon 51 [14]. Despite a lower dose injected ICV, tcDNA exhibited slightly higher exon skipping levels than PMO in the brain. Interestingly though, the distribution pattern was very similar between the two chemistries and mirrored our prior findings, indicating higher skipping in the hippocampus compared to the cerebellum and cortex for both chemistries. This further reinforces the consistency in distribution patterns following ICV administration of ASOs in mouse brains.

A notable aspect of our investigation lies in the unexpected lack of substantial protein rescue despite achieving efficient exon 53 skipping. The underlying reasons remain unclear, leaving us to hypothesize potential mechanisms. We have previously and repeatedly reported a discrepancy between the exon skipping levels and protein restoration levels in the *mdx52* mouse model, notably after exon 51 skipping [13,19] which may be due to the known *DMD* transcript imbalance [26,27]. However, this discrepancy is generally around two- to four-fold and it, therefore, does not explain how 25% of measured exon skipping in the hippocampus, for example, translates into less than 2% of protein restoration. It is possible to hypothesize that, in addition to the precise skipping of exon 53, additional exon(s) along the *Dmd* transcript are also eliminated by the cocktail of ASOs, leading to out-of-frame transcripts which are unable to give rise to any protein expression. We performed PCR of a longer fragment around exon 53, notably from exons 47 to 58, and did not detect the presence of such transcripts, but we cannot exclude that exons outside of this region are eliminated. Further investigations including RNA-Seq analysis would be required. An alternative explanation could be the stability of the restored protein, considering the potential scenario where the absence of exons 52 and 53 results in a less stable protein compared to the one encoded by the previously characterized Δ51–52 transcript [13]. This is not particularly supported by clinical data that indicate that DMD patients treated with the exon 53 skipping ASO viltolarsen are among the ones displaying the highest percentage of dystrophin rescue in muscles (mean of 5.9%) [28] compared with other exons skipped like exon 51 or 45, nor is supported by the BMD with these naturally occurring DMD deletions. Yet, this does not exclude a possible difference in stability of these proteins in the CNS, which remains unknown. It is possible that the produced protein is less stable than the protein lacking exon 51 and 52 and, therefore, does not accumulate or accumulates slower. Further exploration into the discrepancies between exon skipping levels and protein expression is warranted to unravel the intricacies of exon 53’s skipping impact on protein restoration.

Nonetheless, our study provides the valuable insight that currently the *mdx52* mouse model is not suitable to investigate exon 53 skipping to concurrently restore both Dp427 and Dp140. This model, while useful for certain investigations, indeed proved inadequate for exon 53 skipping exploration because of its difference with the human exon 53 skipping. As a potential alternative, the hDMDdel52/*mdx* mice could be considered. This is a mouse model where the human *DMD* gene with a deletion of exon 52 is integrated in the mouse genome in an *mdx* background (nonsense mutation in mouse *Dmd* exon 23). However, one should keep in mind that mouse Dp140 is still expressed in the brain of this model (the *mdx* solely lacking Dp427). A more suitable model would necessitate the crossbreeding of hDMDdel52 with the *mdx52* model or the DMD-null mice, created by deleting the entire *Dmd* genomic region [29]. As the hDMDdel52 genes are integrated on mouse chromosome 5, while the *mdx* mutation is located on the X chromosome, this would involve extensive and time-consuming breeding schedules and large numbers of mice. However, the resulting models would allow the assessment of human exon 53 skipping to study the impact of restoring both Dp427 and Dp140 in the CNS. Investigating this postnatal restoration indeed remains critical to better understand DMD’s neurological aspects and pave the way for comprehensive therapeutic strategies. Considering that Dp140 is expressed in the fetal brain, with high expression in the early to mid-fetal stages [30], future studies should aim at restoring this isoform as early as possible. Yet, previous results from Hashimoto and colleagues suggested that even low restoration of Dp140 in adult *mdx52* mice could have a therapeutic effect [31]. The journey towards addressing the central manifestations in DMD remains challenging but promises to unravel new insights critical for advancing the care of those affected by this complex disorder.

## Figures and Tables

**Table 1:** List of all ASOs used in the study. ASOs that were selected for combination studies are highlighted in color: blue for region 1, pink for region 2, green for region 3 and yellow for region DS.

CHEMISTRY	REGION	NAME	TARGET POSITION	ABBREVIATED NAME	TARGET SEQUENCE
**tcDNA**	PRE-SCREENING	tcDNA +27+41	+27+41		GAGGTTCAAGAACAG
		tcDNA +33+47	+33+47		CAAGAACAGCTGCAG
		tcDNA +36+50	+36+50		GAACAGCTGCAGAAC
		tcDNA +38+52	+38+52		ACAGCTGCAGAACAG
		tcDNA +40+54	+40+54		AGCTGCAGAACAGGA
		tcDNA +41+55	+41+55		GCTGCAGAACAGGAG
		tcDNA +42+56	+42+56		CTGCAGAACAGGAGA
		tcDNA +43+57	+43+57		TGCAGAACAGGAGAC
		tcDNA +44+58	+44+58		GCAGAACAGGAGACA
		tcDNA +46+60	+46+60		AGAACAGGAGACAAC
		tcDNA +59+73	+59+73		ACAGTTGAATGAAAT
		tcDNA +73+87	+73+87		TGTTAAAGGATTCAA
	R1	tcDNA +33+52	+33+52		CAAGAACAGCTGCAGAACAG
		tcDNA +36+55	+36+55	tcDNA_R1a	GAACAGCTGCAGAACAGGAG
		tcDNA +39+58	+39+58		CAGCTGCAGAACAGGAGACA
		tcDNA +42+61	+42+61		CTGCAGAACAGGAGACAACA
		tcDNA +45+64	+45+64	tcDNA_R1b	CAGAACAGGAGACAACAGTT
		tcDNA +56+75	+56+75		ACAACAGTTGAATGAAATGT
	R2	tcDNA +69+88	+69+88		GAAATGTTAAAGGATTCAAC
		tcDNA +72+91	+72+91	tcDNA_R2	ATGTTAAAGGATTCAACACA
		tcDNA +75+94	+75+94		TTAAAGGATTCAACACAATG
		tcDNA +78+97	+78+97		AAGGATTCAACACAATGGCT
		tcDNA +100+120	+100+120		AAGCTAAGGAAGAAGCCGAA
	R3	tcNDA +125+144	+125+144		TCATAGGACAGGTCAGAGGC
		tcDNA +128+147	+128+147		TAGGACAGGTCAGAGGCAAG
		tcDNA +131+150	+131+150	tcDNA_R3	GACAGGTCAGAGGCAAGCTT
		tcDNA +134+153	+134+153		AGGTCAGAGGCAAGCTTGAC
	DS	tcDNA +5−15	+5−15	tcDNA_DS	CCAAGgttagtgtcaagcat
		tcDNA +3−18	+3−18		AAGgttagtgtcaagcatat
		tcDNA +1-20	+1-20		Ggttagtgtcaagcatatct
					
**PMO**	R1	PMO +36+60	+36+60	PMO_R1a	GAACAGCTGCAGAACAGGAGACAAC
		PMO +43+67	+43+67	PMO_R1b	GCAGAACAGGAGACAACAGTTGAAT
	R2	PMO +69+98	+69+98	PMO_R2	GAAATGTTAAAGGATTCAACACAAT
	R3	*No PMO for this region*			
	DS	PMO +9−16	+9−16	PMO_DS	GAAACCAAGgttagtgtcaagcata
					
**MOE**		MOE +9+26	+9+26		TTCAGATTCAGTGGGATGTG
	R1	MOE +28+45	+28+45	MOE_R1	GGTTCAAGAACAGCTGCA
	R2	MOE +79+98	+79+98	MOE_R2	TCAACACAATGGCTGGAAGCT
		MOE +83+102	+83+102		CAACACAATGGCTGGAAGCT
		MOE +84+103	+84+103		TCAACACAATGGCTGGAAGC
	R3	MOE +135+154	+135+154	MOE_R3	TCAGAGGCAAGCTTGACTCA
	DS	MOE +5−15	+5−15	MOE_DS	CCAAGgttagtgtcaagcat

## Data Availability

The raw data that support the findings of this study are available from the corresponding author upon reasonable request.

## Supplementary Materials

The following supporting information can be downloaded at: https://www.mdpi.com/article/10.3390/biomedicines11123243/s1. Figure S1. Screening of tcDNA53 target sequences; Figure S2. In vitro analysis of exon 53 skipping levels induced by PMO-ASOs; Figure S3. In vitro analysis of exon 53 skipping levels induced by 2′MOE-ASOs; Figure S4. Quantification of Dp140 protein restoration by Western blot in hippocampus 10 weeks after ICV administration; Figure S5. Comparison of mouse and human exon 53 splicing regulatory signals using the Human Splicing Finder software. Figure S6. Quantification of Dp427 and Dp140 after ICV injection of 4 tcDNA-ASO. Figure S7. Comparison of mouse and human exon 53 splicing regulatory signals using the Human Splicing Finder software.
