# Supplementary material for: Networking to Optimize Dmd exon 53 Skipping in the Brain of mdx52 Mouse Model
Source: Biomedicines. 2023 Dec 7;11(12):3243. doi: 10.3390/biomedicines11123243 (PMC10741439; doi:10.3390/biomedicines11123243)
Supplement: Supplementary file 1 [file biomedicines-11-03243-s001.zip › biomedicines-2646553-supplementary.pdf]

Supplementary material

Networking to optimize *Dmd* exon 53 skipping in the brain of *mdx52* mouse model

Mathilde Doisy[1], Ophélie Vacca[1], Claire Fergus[2], Talia Gileadi[3,7], Minou Verhaeg[4], Amel Saoudi[1,5], Thomas Tensorer[6], Luis Garcia[1], Vincent P. Kelly[2], Federica Montanaro[3,7], Jennifer E. Morgan[3,7], Maaike van Putten[4], Annemieke Aartsma-Rus[4], Cyrille Vaillend[5], Francesco Muntoni[3,7] and Aurélie Goyenvalle[1, *]

1 Université Paris-Saclay, UVSQ, Inserm, END-ICAP, 78000 Versailles, France
2 School of Biochemistry & Immunology, Trinity Biomedical Sciences Institute, Trinity College Dublin,
Dublin D02 R590, Ireland
3 Dubowitz Neuromuscular Centre, UCL Great Ormond Street Institute of Child Health, 30 Guildford Street, London WC1N 1EH, UK.
4 Department of Human Genetics, Leiden University Medical Center, 2333ZA Leiden, The Netherlands.
5 Université Paris-Saclay, CNRS, Institut des Neurosciences Paris-Saclay, 91400 Saclay, France
6 SQY Therapeutics-Synthena, UVSQ, 78180 Montigny le Bretonneux, France.
7 National Institute for Health Research, Great Ormond Street Institute of Child Health Biomedical Research Centre, University College London, London, UK

* Correspondence: aurelie.goyenvalle@uvsq.fr

A

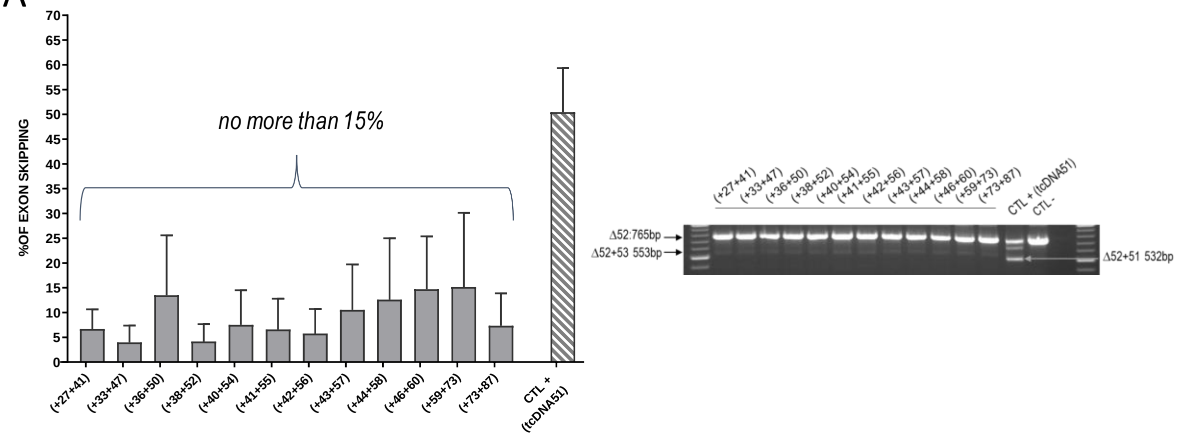

B

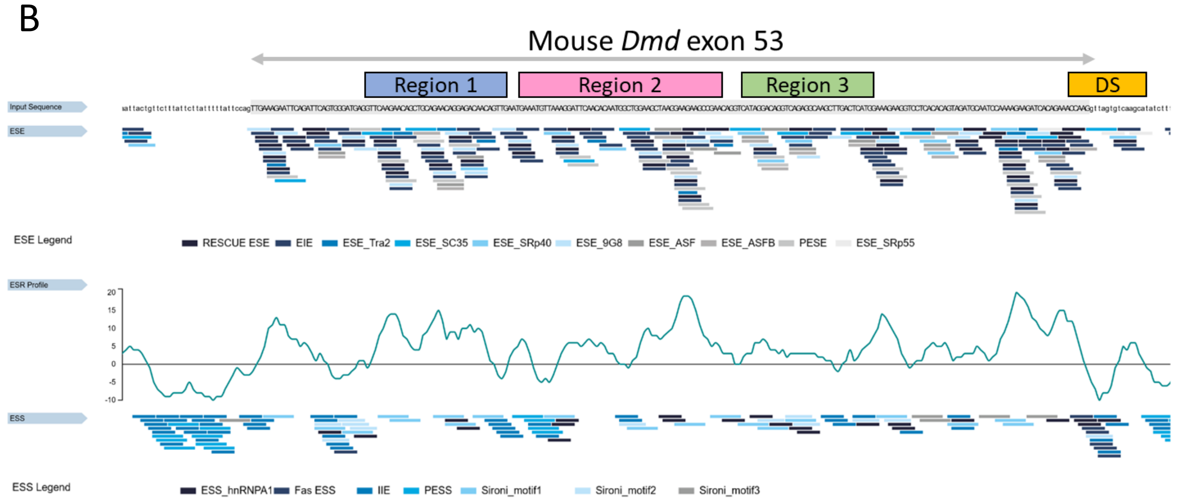

C

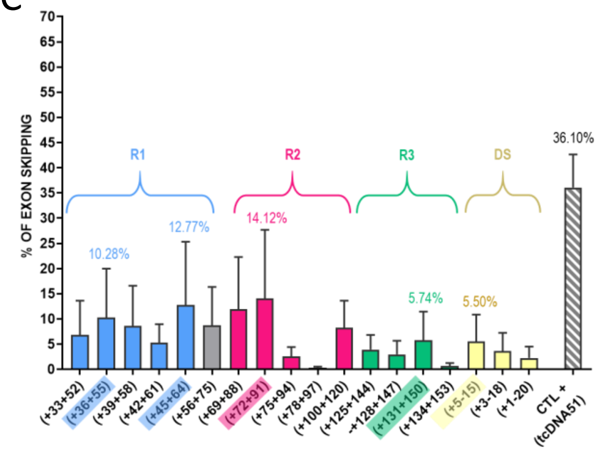

REGION	NAME	TARGET POSITION	MEAN %SKIP (n=3)	
R1	tcDNA +33+52	+33+52	6.83	
	tcDNA +36+55	+36+55	10.28	tcDNA_R1a
	tcDNA +39+58	+39+58	8.65	
	tcDNA +42+61	+42+61	5.31	
	tcDNA +45+64	+45+64	12.77	tcDNA_R1b
	tcDNA +56+75	+56+75	8.76	
R2	tcDNA +69+88	+69+88	11.94	
	tcDNA +72+91	+72+91	14.12	tcDNA_R2
	tcDNA +75+94	+75+94	2.52	
	tcDNA +78+97	+78+97	0.34	
	tcDNA +100+120	+100+120	8.29	
R3	tcNDA +125+144	+125+144	3.94	
	tcDNA +128+147	+128+147	2.97	
	tcDNA +131+150	+131+150	5.74	tcDNA_R3
	tcDNA +134+153	+134+153	0.63	
DS	tcDNA +5-15	+5-15	5.5	tcDNA_DS
	tcDNA +3-18	+3-18	3.64	
	tcDNA +1-20	+1-20	2.26	

Figure S1. Screening of tcDNA53 target sequences. (A) Quantification of exon 53 skipping levels by RT-PCR after transfection of *H2K-mdx52* mouse myotubes with 15-mer tcDNA53. Results are expressed as means ± SEM; n=3 transfections compared to tcDNA51 exon 51 skipping level (CTL+ 50%). Results reveal no more than 15% of exon 53 skipping. (B) Analysis of mouse exon 53 splicing regulatory signals using the Human Splicing Finder software (https://hsf.genomnis.com), and localisation of the regions named Region 1, 2, 3 and DS. (C) Quantification of exon 53 skipping levels by RT-PCR after transfection of *H2K-mdx52* mouse

myotubes with 20-mer tcDNA53. Results are expressed as means ± SEM; n=3 transfections compared to tcDNA51 exon 51 skipping level (CTL+ 36%). The best ASOs were selected for each region and are highlighted in the table on the right panel.

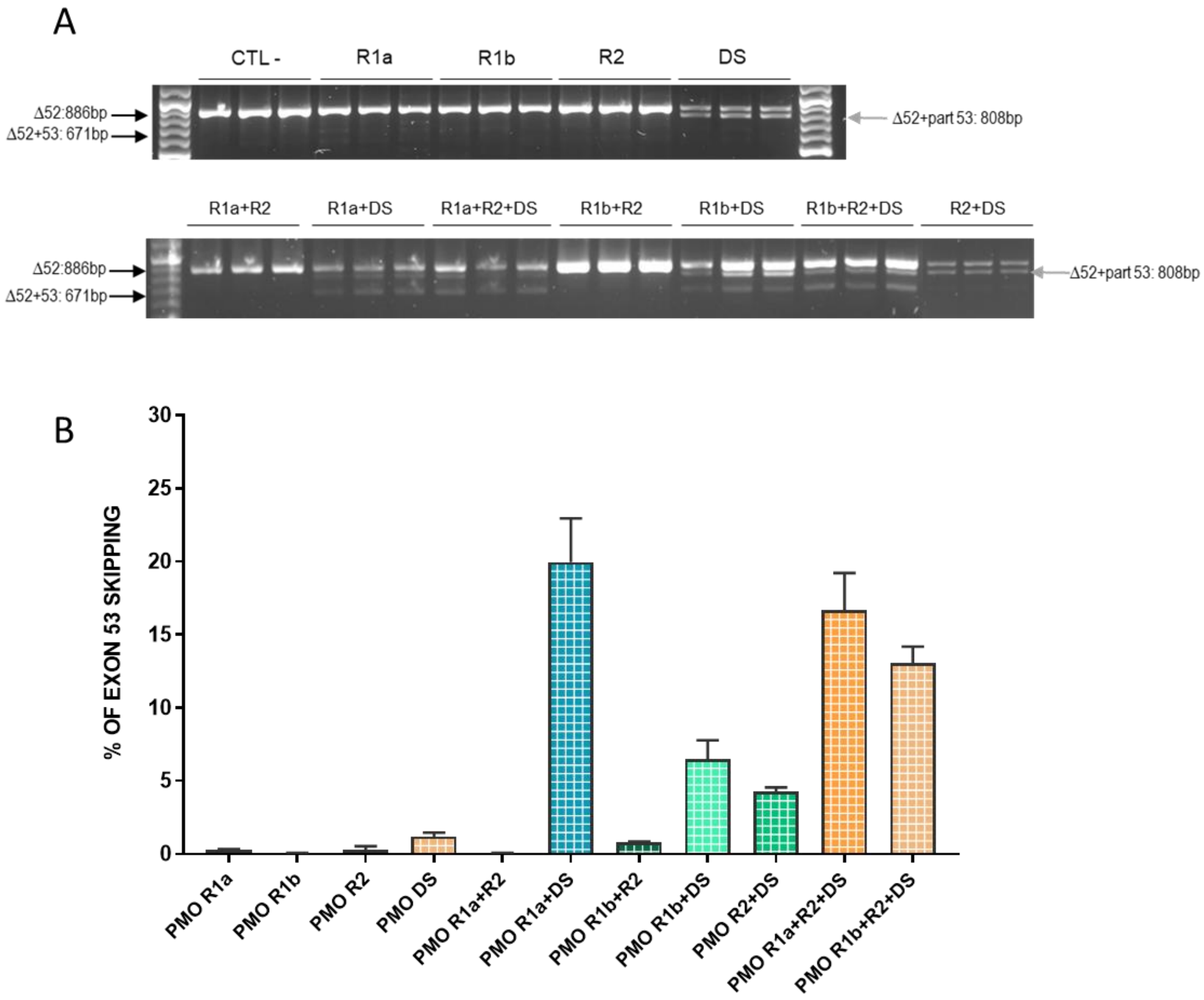

Figure S2. ***In vitro* analysis of exon 53 skipping levels induced by PMO-ASOs.** (A) Visualization on gel electrophoresis and (B) quantification of exon 53 skipping levels after RT-PCR of C2C12 cells transfected with different PMO-ASO targeting mouse exon 53. Results are expressed as means ± SEM; n=3 transfections. The intermediate band at 808bp between the unskipped and the exon 53 skipped band observed in particular with the PMO DS corresponds to the partial skipping of exon 53 (only 78 nt are eliminated due to the presence of a cryptic donor splice site), as previously described by Mitrpant et al., 2009.

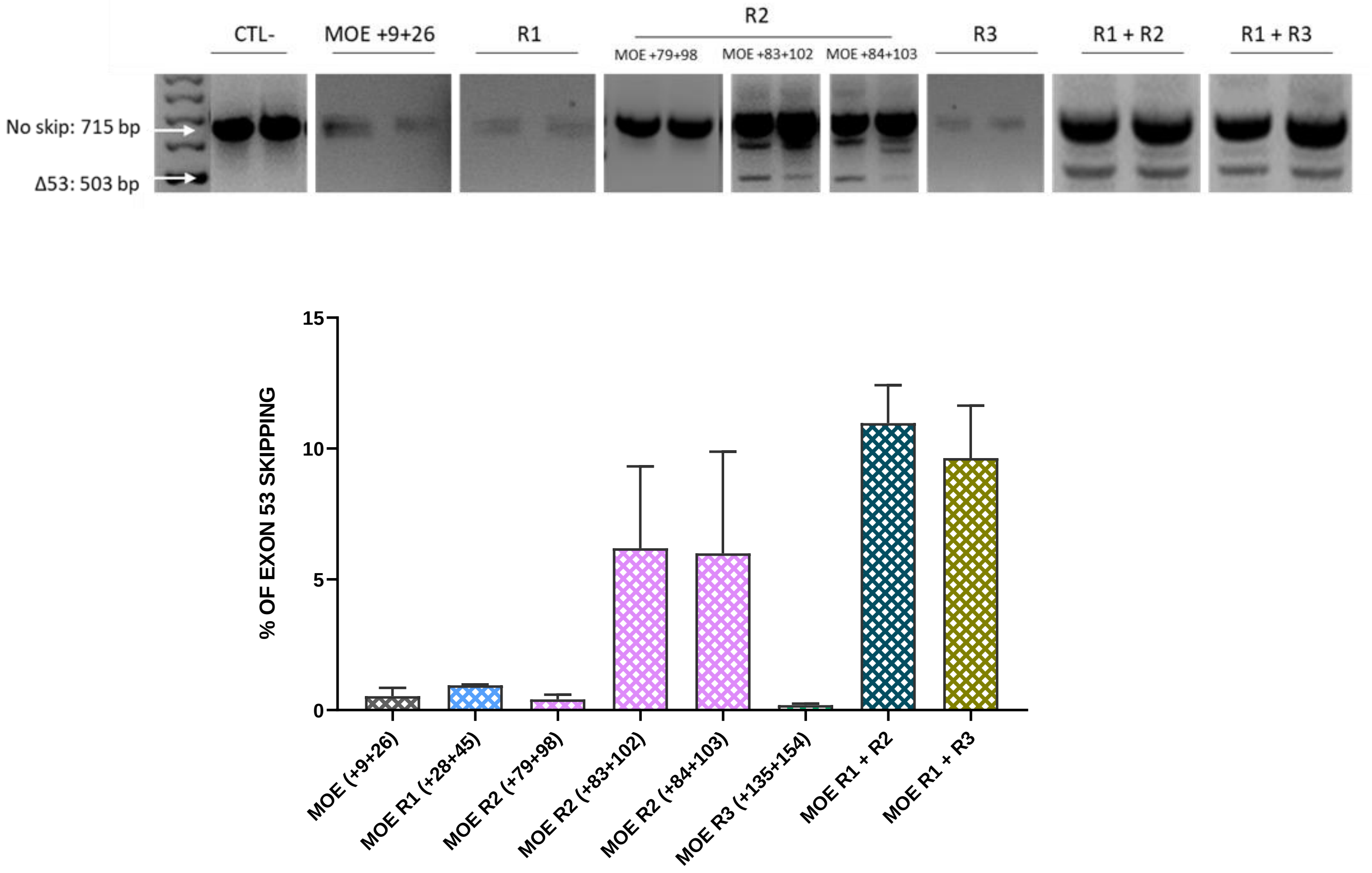

Figure S3. ***In vitro*** **analysis of exon 53 skipping levels induced by 2′MOE-ASOs.** (A) Visualization on gel electrophoresis and (B) quantification of exon 53 skipping levels after RT-PCR of C2C12 cells transfected with different 2′MOE-ASO targeting mouse exon 53.

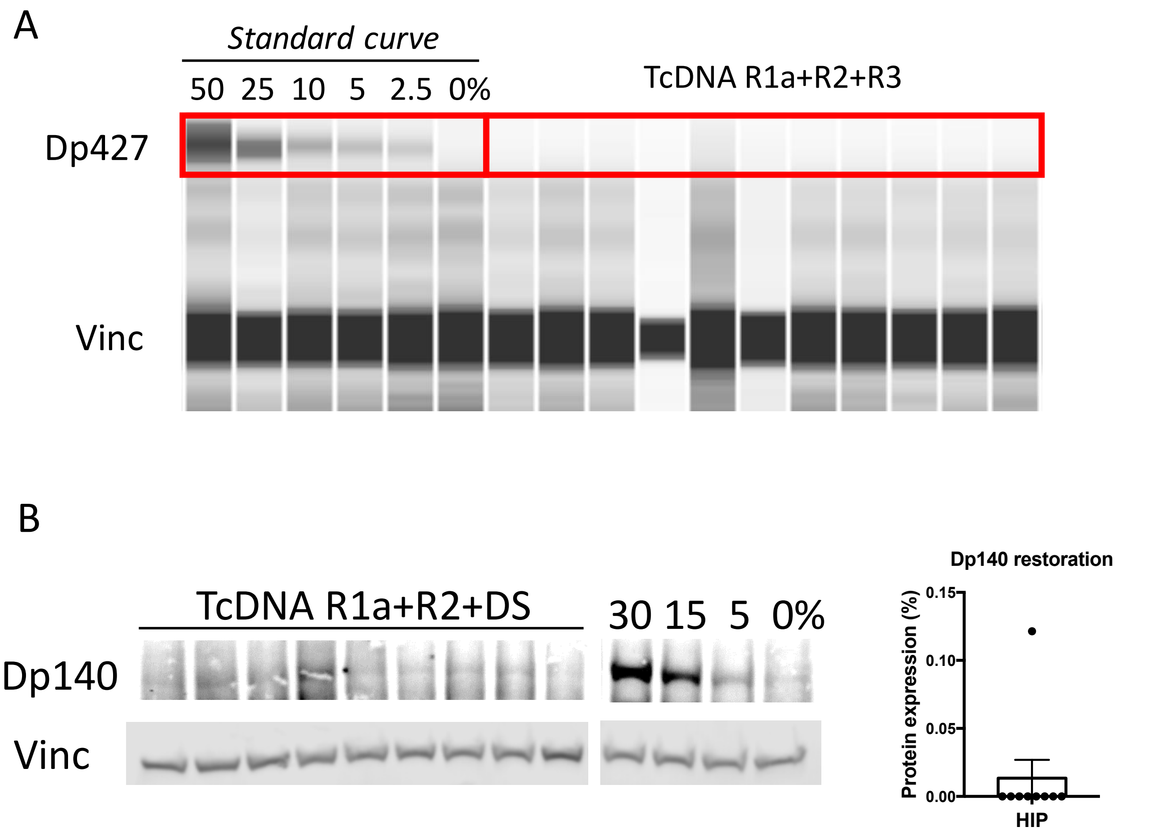

Figure S4. A) Capillary western immunoassay (Jess) to confirm the barely detectable expression of Dp427 in hippocampus 10 weeks after ICV administration. A 6-point standard curve made of 0, 2.5, 5, 10, 25 and 50% of WT lysate (mixed with *mdx52* lysate) was loaded for quantification. Vinculin (Vinc) was used as control for normalization. B) Quantification of Dp140 protein restoration by western blot in hippocampus (Hip) 10 weeks after ICV administration. A 4-point standard curve made of 0, 5, 15 and 30% of WT lysate (mixed with *mdx52* lysate) was loaded for quantification. Vinculin was used as control for normalization.

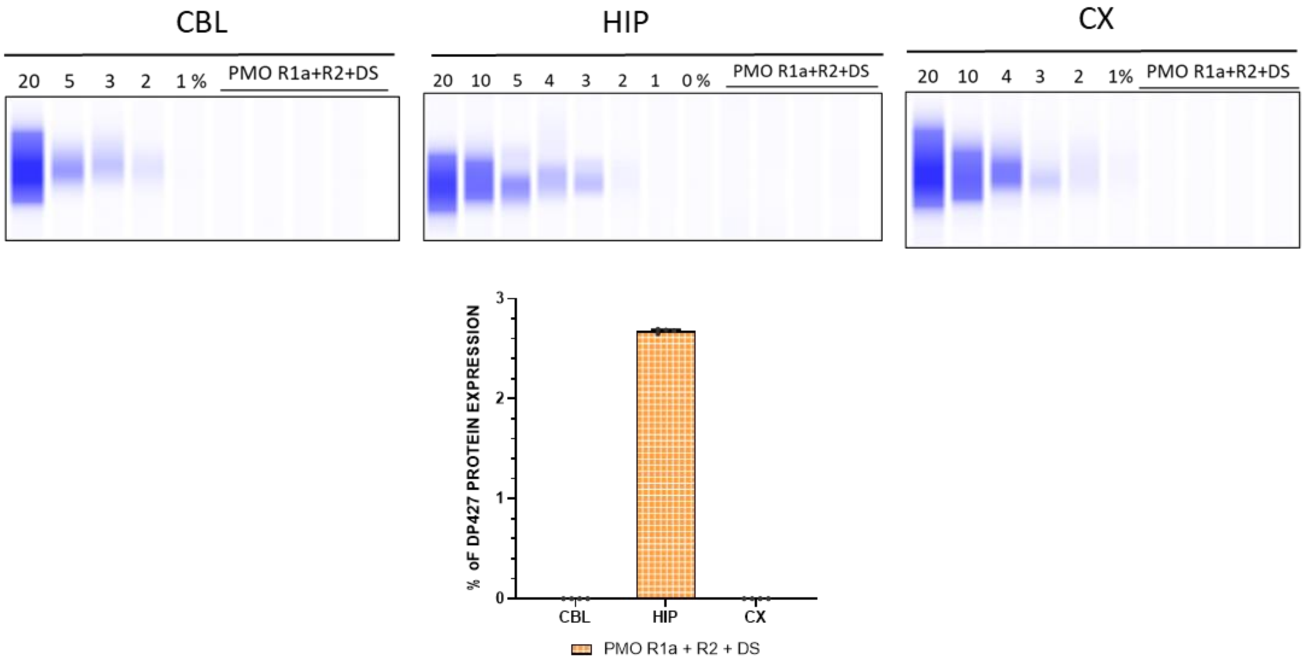

Figure S5. Quantification of Dp427 restoration in cerebellum, hippocampus and cortex 8 weeks after ICV administration of PMO (R1a+R2+DS) by capillary western immunoassay (Wes). A 5 to 8-point standard curve made of WT lysate mixed with *mdx*52 lysate was loaded for quantification. Cerebellum (CBL), hippocampus (HIP), cortex (CX).

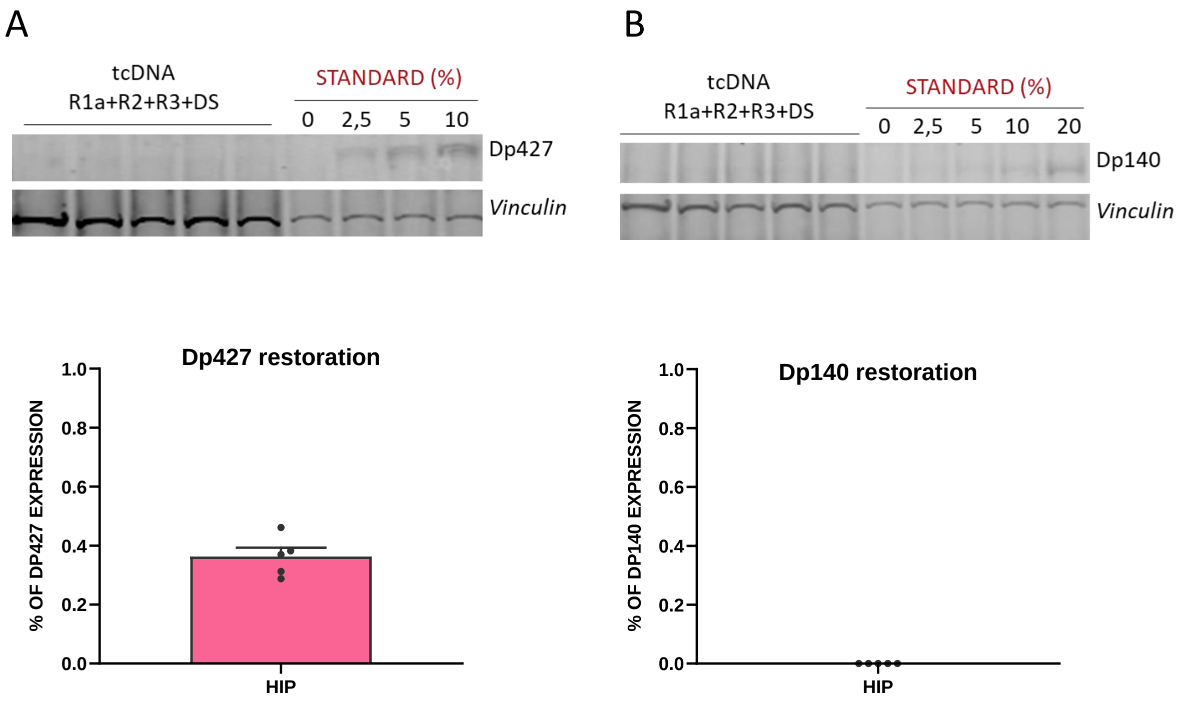

Figure S6. Quantification of Dp427 and Dp140 after ICV injection of 4 tcDNA-ASO. Quantification of Dp427 (A) and Dp140 (B) restoration in hippocampus following ICV injection of tcDNA R1a+R2+R3+DS in *mdx52* mice. A 4-point standard curve made of 0, 2.5, 5 and 10% of WT lysate (mixed with *mdx*52 lysate) was loaded for quantification. Vinculin was used as control for normalization. Results are expressed as means ± SEM; n=5 treated *mdx52* mice.

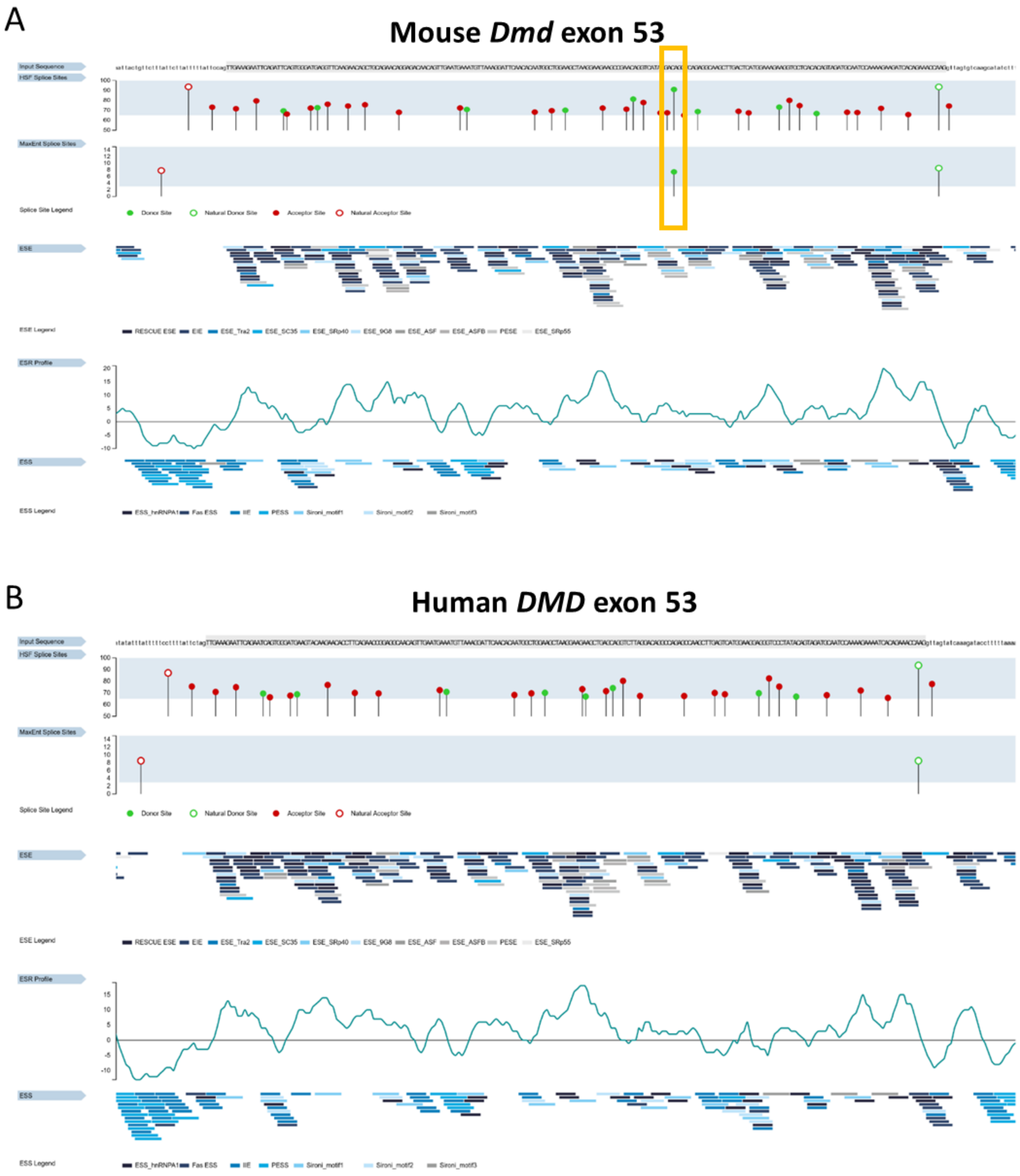

Figure S7: Comparison of mouse and human exon 53 splicing regulatory signals using the Human Splicing Finder software. (A) Analysis of mouse exon 53 splicing regulatory signals using the Human Splicing Finder software (https://hsf.genomnis.com). (B) Analysis of human exon 53 splicing regulatory signals using the Human Splicing Finder software. The entire sequence of exon 53 has been entered (212 nt) + 50 nt of intronic sequence upstream and downstream of the exon (i.e. a sequence of 312 nt in total) for the analysis. The orange rectangle highlights the presence of a cryptic donor splice site in mouse exon 53 (at position 134).
